# Hybrid approach in sacral sore management with maggot debridement therapy and flap reconstruction

**DOI:** 10.1016/j.jpra.2023.12.006

**Published:** 2023-12-06

**Authors:** Marvin Man Ting Chung, Samuel Yan Jin Fang, Alex Pat Chung Yeung, Wai Fung Kwong, Yuk Fai Lui, Wing Yuk Ip

**Affiliations:** a Department of Orthopaedics & Traumatology, University of Hong Kong, Hong Kong; b Department of Orthopaedics & Traumatology, Queen Mary Hospital, Hong Kong

**Keywords:** Sacral sore, Decubitus ulcer, Pressure sore, Maggot debridement, Flap reconstruction

## Abstract

Sacral sore is a common problem in patients with spinal cord injury. It leads to prolonged hospitalization and recurrent infections which might require repeated surgery to treat. Flap reconstruction allows soft tissue coverage of sacral sore under the premise of infection-free wound base. Maggot debridement therapy (MDT) has been described as an alternative non-surgical management as opposed to the traditional surgical debridement in case of infected sore, reducing number of surgeries under anaesthesia. However, MDT and surgery are not mutually exclusive. In this article we describe a hybrid approach combining MDT and flap reconstruction with multi-disciplinary effort in management of sacral sore, which accelerates wound healing and prevents morbidities, while lowering the risks associated with repeated surgical debridement at the same time.

## Introduction

Pressure sores are common problems in spinal cord injury patients with paraplegia or tetraplegia, resulting in recurrent infections, prolonged hospitalization, repeated surgery, high treatment cost, and even mortalities.^1^ Prevention of pressure sore is always the best treatment, with majority being avoidable with regular turning, repositioning and good nursing care.^2^ However, in cases where tissue necrosis and secondary infection has set in, step-wise multi-disciplinary care with consideration of patients’ co-morbidities and risk factors is required. Flap reconstruction for pressure sore coverage allows reliable closure of raw area, prevents hospitalization and decreases morbidities. However, many pressure sores were infected in various degrees, requiring multiple attempts of surgical debridement under anaesthesia, with accompanied higher risk of anaesthesia-related and peri‑operative complications.

Non-surgical debridement modalities such as maggot debridement therapy (MDT) have therefore increasingly achieved attention for treatment of pressure sores, which involves biological debridement by sterile larvae of *Lucilia sericata*.^3^ MDT involves application of maggots feeding on necrotic tissue and promoting granulation tissue growth, where maggots are subsequently removed after 2–3 days with thorough irrigation before a new batch of maggots is applied as another cycle of treatment. MDT allows patients to receive debridement of necrotic tissues without undergoing multiple surgery, thereby reducing surgical risks and peri‑operative complications, especially in patients with poor co-morbidities.

While there are reports of using MDT as conservative management of pressure sore, and flap reconstruction for pressure sore coverage, combination of the two approaches and a streamlined workflow has not been reported widely in literature. In this article we illustrate our center's hybrid approach in management of sacral sore through an illustrative case, incorporating use of MDT in Biobag fashion and flap reconstruction to effectively close the pressure sore in a single operation. This effectively balances the risk of repeated surgical debridement under anaesthesia and adequate infection clearance, and expedites soft tissue coverage in these medically-vulnerable individuals.

### Case illustration

**Figure 1:**
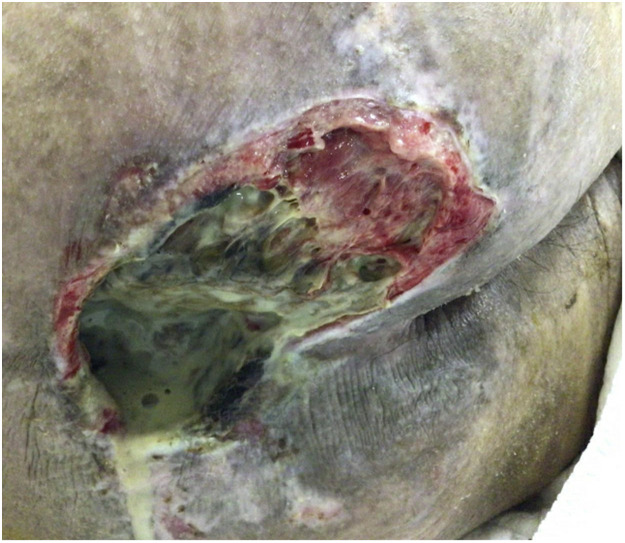
Infected sacral sore with purulent discharge.

A 45-year-old male suffered from fall with L1 vertebral burst fracture and paraplegia. Despite posterior decompression with instrumented lumbar fusion, only partial recovery to lower limb function was gained. He developed a 9 cm sacral sore during rehabilitation, which became infected later with greenish purulent discharge (Figure 1), requiring surgical debridement under general anesthesia. Unhealthy skin and slough superficial to sacrum were removed but radical debridement of sacrum bone was not performed at this stage to prevent dural tear and cerebrospinal fluid leakage, which should only be performed in the same setting with flap coverage.

**Figure 2:**
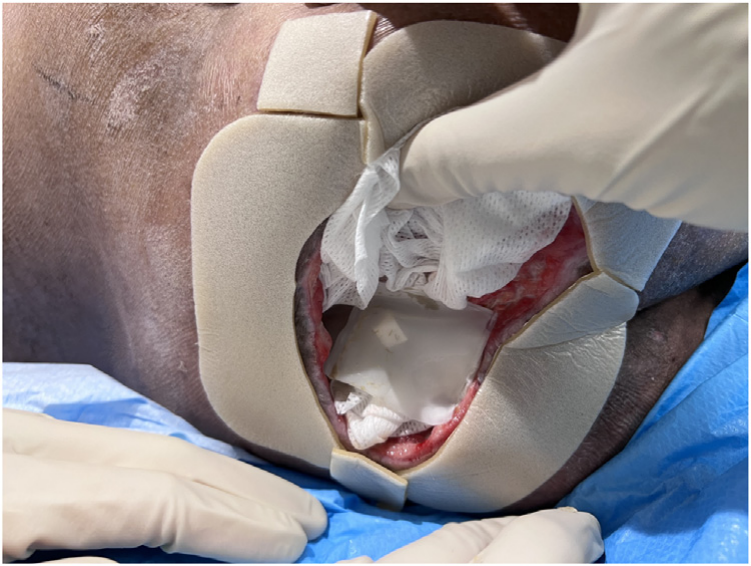
MDT application in a Biobag fashion.

He was put on regular dressing and appropriate antibiotics (piperacillin/tazobactam) for coverage of *Pseudomonas aeruginosa*. Despite that there was persistent discharge and slimy slough at base. To achieve better wound bed without repeated surgical debridement under anesthesia, we opted for MDT in Biobag fashion (Medifly, Cuprina Wound Care Solutions, Singapore) (Figure 2).

**Figure 3:**
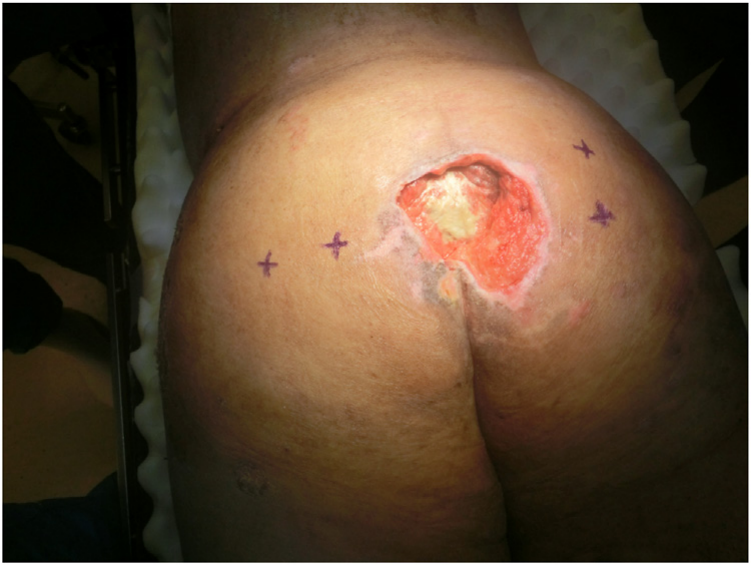
Clean sacral sore after maggot debridement therapy and before flap reconstruction.

**Figure 4:**
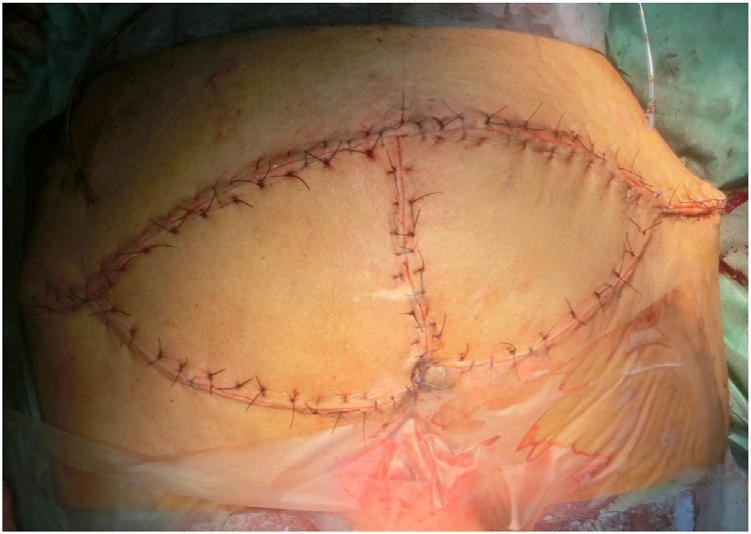
Gluteus maximus perforator-based V-Y fasciocutaneous advancement flap.

**Figure 5:**
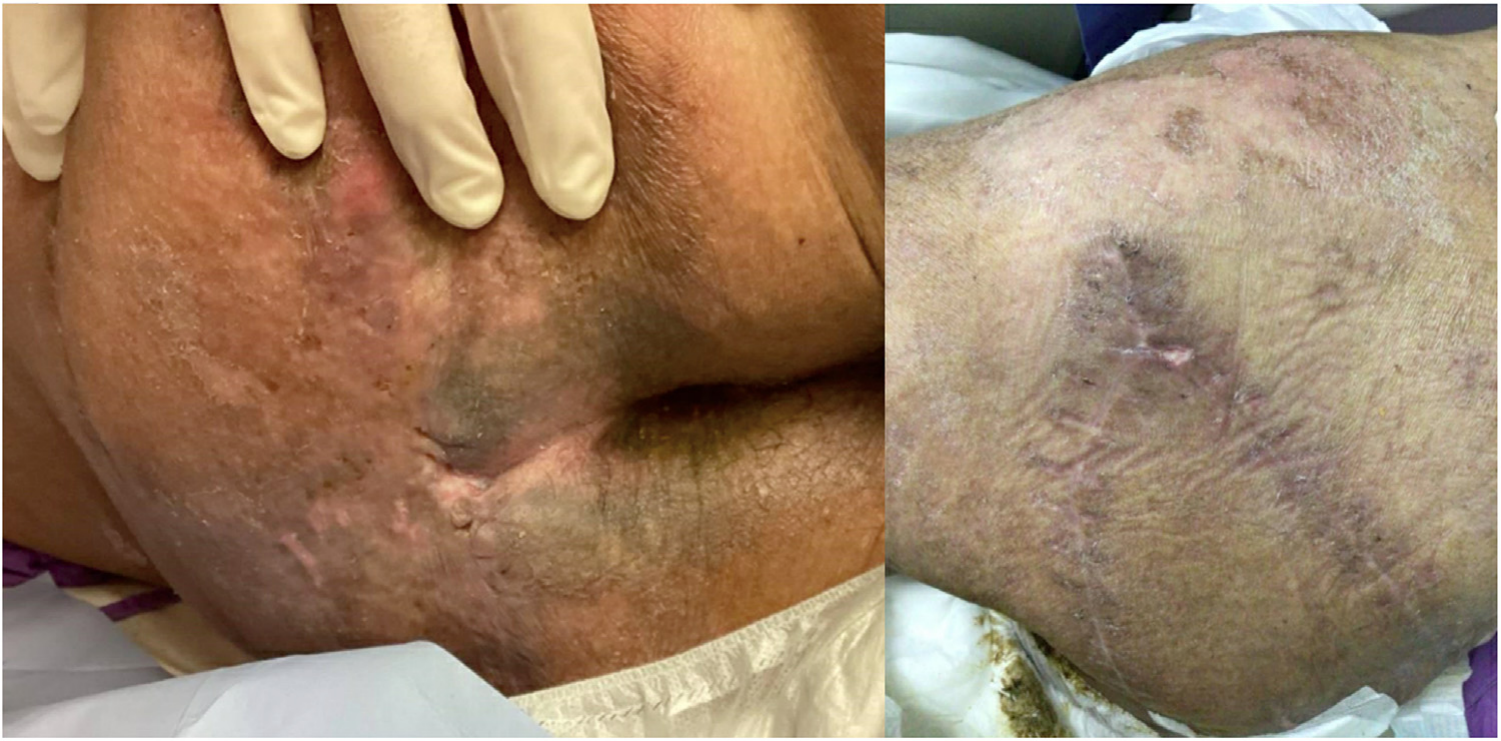
Healed flap for sacral sore coverage.

After two cycles of MDT, better wound bed was achieved without purulent discharge and was deemed ready for flap coverage procedure (Figure 3). Gluteus maximus perforator-based V-Y fasciocutaneous advancement flap was utilized (Figure 4). Further debridement of slough, smoothening of sacral bony prominences and excision of subcutaneous scar tissue were performed to achieve healthy vascularized tissue bed before medial advancement of flap towards midline. The sore eventually healed well (Figure 5).

### Role of MDT in pressure sore management

Maggot secretions demonstrate antimicrobial properties and also promote healing through the induction and amplification of inflammatory cytokines and enzymes. Compared to conventional therapy, higher rate of complete debridement and granulation tissue formation, better healing rate and a significantly shorter time to healing were seen in MDT-treated wounds.^4^

No major adverse events or complications have been reported with use of MDT in the literature, although some patients have reported increased ulcer-related discomfort and pain while on MDT,^5^ however this is not a major concern for pressure sores as they are neuropathic ulcers. Despite reported high acceptance rates among caregivers and patients,^6^ appearance of free-ranging maggots on wounds might cause uneasiness to patients, other patients in adjacent beds in a large ward, and nursing care providers, with chance of maggot escape and incomplete removal in severely undermined wounds. Maggots placed in a Biobag facilitate nursing care and reduce rate of maggot escape, without compromise of debridement efficiency.^7^

### Nursing management of MDT

In our institution we collaborate with specialty wound nurses for MDT application. They provide critical assessment to clinical environment for maggot application, wound characteristics, and an all-round patient assessment including psychological aspects.

Prior to application of MDT, it has been suggested that antiseptic solution be stopped for wound dressing 2 days before MDT which may be harmful to maggot activity.^8^ Hydrocolloid dressing is cut and contoured around wound edge to avoid skin irritation before applying maggots. A dry gauze is secured onto hydrocolloid padding using transparent semi-permeable membrane dressing in a matching pattern.^8^ The central porous part is not covered to allow oxygen exchange for maggots and allow drainage of necrotic wound exudates.

After MDT application, dressing is checked every 4–6 h for loosening or soiling. Frequent clinical assessment of systemic allergic reaction and local adverse effect, and psychological assessment of patient's coping ability is required, together with education to caregivers and other nursing staff who may be unfamiliar to MDT.

Specialty wound nurses have frequent communication with surgeons regarding when to initiate MDT, reviewing indications constantly and discussing end points of MDT. There are two end points considered in MDT, the first being successful eradication of slough and infected tissue, with healthy granulation tissue formation or even re-epithelialization, where MDT will not provide additional benefit compared to traditional dressing; and the second being failure of treatment with residual soft tissue defect or infection, where higher levels of reconstructive ladder or further debridement need to be considered respectively.

### Role of flap reconstruction in sacral sore management

Despite advantages of MDT in avoiding repeated surgery, continuing long repeated cycles of MDT will likely yield less marginal gain in wound healing with time. To accelerate wound healing and coverage, early flap reconstruction is considered in our hybrid approach of sacral sore management once the MDT end point has been reached and no gross infection is evident.

In pressure sores, primary closure and skin graft have high failure rates due to wound breakdown and intolerability towards repeated shearing respectively.^9^ Locoregional flap reconstruction remains as mainstay of treatment. Fasciocutaneous flaps carry less donor site morbidity, while myocutaneous flaps confer increased resilience to infection and added muscle bulk that obliterates dead space in deeper defects. Nonetheless, there is no reported difference in success between the two,^10^ and fasciocutaneous flaps remains the first line choice in our practice. Our choice of advancement flap is based on gluteus maximus muscle with gluteal perforators, which is advanced towards the midline in V-Y fashion. Unilateral advancement can be performed for small defects, but most are large enough to warrant bilateral advancement.

## Conclusion

Both MDT and flap reconstruction are useful tools for pressure sore management, but a hybrid approach combining the two in multi-disciplinary setting would further accelerate wound healing in a safe and effective manner. Careful patient selection, intensive pre-operative optimization, meticulous wound assessment, correct timing of MDT application and individualized treatment strategy are crucial factors in improving patient outcome.

## Declaration of Competing Interest

All authors declare no conflict of interest.

## References

[1] Kruger E.A. (2013). Comprehensive management of pressure ulcers in spinal cord injury: Current concepts and future trends. J Spinal Cord Med.

[2] Black J.M. (2011). Pressure ulcers: avoidable or unavoidable? Results of the national pressure ulcer advisory panel consensus conference. Ostomy Wound Manage.

[3] Shamloul G., Khachemoune A. (2023). Reappraisal and updated review of maggot debridement therapy in chronic lower extremity ulcers. Int J Dermatol.

[4] Wilasrusmee C. (2014). Maggot therapy for chronic ulcer: a retrospective cohort and a meta-analysis. Asian J Surg.

[5] Mudge E. (2014). A randomized controlled trial of larval therapy for the debridement of leg ulcers: results of a multicenter, randomized, controlled, open, observer blind, parallel group study. Wound Repair Regen.

[6] Marineau M.L. (2011). Maggot debridement therapy in the treatment of complex diabetic wounds. Hawaii Med J.

[7] Blake F.A. (2007). The biosurgical wound debridement: Experimental investigation of efficiency and practicability. Wound Repair Regen.

[8] DeVesty G.B.M.P.-C. (2018). J. R. B., Diabetic foot ulcer: Biologic debridement. CINAHL Nursing Guide.

[9] Kenneweg K.A., Welch M.C., Welch P.J. (2015). A 9-year retrospective evaluation of 102 pressure ulcer reconstructions. J Wound Care.

[10] Thiessen F.E. (2011). Flap surgery for pressure sores: Should the underlying muscle be transferred or not?. J Plast Reconstr Aesthet Surg.

